# Advance in Targeted Cancer Therapy and Mechanisms of Resistance

**DOI:** 10.3390/ijms242316584

**Published:** 2023-11-22

**Authors:** Patrick Ming-Kuen Tang, Dongmei Zhang

**Affiliations:** 1 State Key Laboratory of Translational Oncology, Department of Anatomical and Cellular Pathology, Prince of Wales Hospital, The Chinese University of Hong Kong, Hong Kong; 2 College of Pharmacy, Jinan University, Guangzhou 510632, China

Drug resistance remains one of the important clinical challenges, making cancer one of the leading causes of death worldwide. It was originally discovered to be an adaptive reaction that occurs gradually in patients receiving conventional treatments due to the genetic heterogeneity of cancer cells. There is ample evidence that the stress of conventional chemotherapy and radiotherapy can gradually activate drug resistance-related genes in cancer cells as a defense mechanism. Due to the target specificity of new clinical drugs, the genetic diversity of cancer cells also helps them avoid targeted therapies.

Growing evidence points to the critical role of the tumor microenvironment in cancer progression, which brings to light its potential on patients with solid cancers based on the success of T cell-based immunotherapy for blood cancers. Unexpectedly, response rates in patients with solid tumors treated with immunotherapy are surprisingly low because the primary resistance mechanisms are unclear. For example, less than 30% of patients with non-small cell lung cancer have a clinical response to PD-1/L1 blockade [1]. Indeed, immune cells are highly dynamic with cancer-type-specific responses in solid tumors [2]. Therefore, a better understanding of the molecular mechanisms of primary and secondary drug resistance may lead to the discovery of effective strategies and novel therapeutic targets to improve clinical outcomes in cancer patients.

Here, we present a Special Issue in partnership with the International Journal of Molecular Sciences as a platform to share the latest findings and novel ideas in clinical and laboratory settings. Following peer-review evaluation, we are pleased to publish a total of 13 papers, including 11 original studies, 1 case report, and 1 review article, in this Special Issue. These high-quality works are produced by clinical scientists and basic researchers around the world. Their content covers multiple disciplines, ranging from laboratory discoveries in experimental cancer models to clinical observations in cancer patients, and encompassing (1) clinical discovery, (2) resistance mechanisms, and (3) novel therapeutics. We hope this collection of papers will inspire researchers to accelerate the development of effective solutions to this important clinical challenge. Below is a brief overview of each study.

**Clinical discovery**

Targeted therapies are designed to inhibit cancer cells in a specific way, offering patients better results and fewer side effects than traditional chemotherapy and radiation therapy. Paradoxically, their therapeutic effect not only inhibits cancer growth but also accelerates the genetic evolution of cancer cells, creating new populations that are resistant to targeted treatments. In this Special Issue, Basse et al. reported novel resistance mechanisms in patients with metastatic lung cancer, with acquired TP53 mutations and Ki67 overexpression observed in cases that are clinically resistant to novel anti-EGFR therapies [3].

Therefore, recent research has attempted to maximize the beneficial efficiency of targeted therapies by identifying patients with suitable biomarkers. Mutations in SF3B1 (subunit 1 of the splicing factor 3b protein complex) were found to be positively associated with poorer response to RG7388-based MDM2-targeted therapy in patients with chronic lymphocytic leukemia [4]. The serpin protein SPINK2 has been suggested as a potent adverse prognostic marker in acute myeloid leukemia, particularly in patients with NPM1 mutations [5]. Furthermore, Gan et al. proposed a non-heme iron-containing enzyme, aspartate β-hydroxylase, as a predictive biomarker for neoadjuvant chemotherapy in patients with advanced gastric cancer [6].

**Resistance mechanisms**

A better understanding of the causative and regulatory mechanisms of drug resistance through in vitro studies or in vivo experimental animal models may lead to the discovery of effective strategies to improve clinical outcomes in cancer treatment.

Martinez et al. studied docetaxel-resistant cell lines in advanced prostate cancer during anti-androgen therapy and taxane-based chemotherapy, and demonstrated that the glucocorticoid receptor acting on docetaxel through a β-catenin-dependent mechanism plays a key role in drug-resistant cancer cells in vitro [7]. The potential contribution of metabolic reprogramming in breast cancer drug resistance was elucidated by analyzing metabolic changes in the doxorubicin-resistant MCF-7 cell line at the protein level using liquid chromatography–mass spectrometry [8]. A comparison of the genetic characteristics of patient tumor samples and normal tissues via bioinformatics analysis revealed the promoting role of the cell cycle-related gene DEPDC1 in glycolysis-related pathways in oral squamous cell carcinoma [9]. In addition, calcium signaling is important in regulating cancer cell apoptosis and may lead to new strategies to overcome drug resistance in the clinical setting [10].

With the advancement of single-cell bioinformatics analysis, new pathogenic mechanisms have been discovered in the tumor microenvironment [11,12], which may also become an effective therapeutic target to overcome cancer drug resistance.

**Novel therapeutics**

Encouragingly, many basic-research-driven inventions have shown impressive anticancer efficiency and safety in in vitro and in vivo experimental cancer models [13]. Brandt et al. indicated that a low-molecular-weight inhibitor of NEDD8-activating enzyme can effectively overcome temozolomide resistance in glioblastoma in vitro [14]. Traditional Chinese medicine is an important source of drug discovery, and its ingredients have been shown to inhibit the growth of multidrug-resistant human cancer cells in vitro and in vivo [15]. Consistent with this notion, the herb-derived natural compounds, polyphyllin I [16] and thymoquinone [17], exhibit anticancer effects on colon cancer and breast cancer, respectively, where polyphyllin I inhibits the growth of cancer cells while thymoquinone suppresses the pathogenic activities of tumor-associated macrophages in vitro.

Gene therapy is also a possible cancer treatment strategy, but its off-target effects on the central nervous system and potential oncogenic activity have largely limited its clinical translation. Kang et al. invented a superparamagnetic iron oxide nanocage, which realized targeted gene therapy based on tumor-specific siRNA and was used to treat osteosarcoma in vivo [18]. Piiper’s group demonstrated that a bifunctional cyclic peptide, CEND-1, shows high tumor selectivity and duration in preclinical tumor models of liver cancer and breast cancer in vivo [19], which may provide a tumor-specific delivery system for anticancer drugs. In another study, it was reported that a virus-free gene therapy system can specifically release genetic modifiers in solid tumors without affecting normal tissues in mice, representing an advanced system for applying gene therapy to cancer patients [20].

**Summary**

Taken together, these papers outline the importance of advances in research into targeted therapies and mechanisms of resistance in cancer and demonstrate the clinical relevance and translational potential of this Special Issue, which may provide insights and inspiration for further research endeavors. Our understanding of cancer drug resistance continues to advance.
